# Functional Lipids and Cardiovascular Disease Reduction: A Concise Review

**DOI:** 10.3390/nu16152453

**Published:** 2024-07-28

**Authors:** Deborah O. Omachi, Alberta N. A. Aryee, John O. Onuh

**Affiliations:** 1 Department of Food and Nutritional Sciences, Tuskegee University, 1200 W. Montgomery Rd, Tuskegee, AL 36088, USA; domachi3100@tuskegee.edu; 2 Food Science and Biotechnology Program, Department of Human Ecology, Delaware State University, 1200 Dupont Highway, Dover, DE 19901, USA; aaryee@desu.edu

**Keywords:** dietary, cardiovascular diseases, biomarkers, supplements, eicosanoids, polyunsaturated fatty acids, chronic diseases, health

## Abstract

Functional lipids are dietary substances that may have an impact on human health by lowering the risk of chronic illnesses and enhancing the quality of life. Numerous functional lipids have been reported to have potential health benefits in the prevention, management, and treatment of cardiovascular disease, the leading cause of death in the United States. However, there is still insufficient and contradictory information in the literature about their effectiveness and associated mechanisms of action. The objective of this review, therefore, is to evaluate available literature regarding these functional lipids and their health benefits. Various studies have been conducted to understand the links between functional lipids and the prevention and treatment of chronic diseases. Recent studies on phytosterols have reported that CLA, medium-chain triglycerides, and omega-3 and 6 fatty acids have positive effects on human health. Also, eicosanoids, which are the metabolites of these fatty acids, are produced in relation to the ratio of omega-3 to omega-6 polyunsaturated fatty acids and may modulate disease conditions. These functional lipids are available either in dietary or supplement forms and have been proven to be efficient, accessible, and inexpensive to be included in the diet. However, further research is required to properly elucidate the dosages, dietary intake, effectiveness, and their mechanisms of action in addition to the development of valid disease biomarkers and long-term effects in humans.

## 1. Introduction

Cardiovascular disease (CVD) is one of the main causes of illness and mortality worldwide [1,2,3]. About 17.8 million people died from CVD in 2017, an increase of 21.1% from 2007, accounting for about one-third of deaths worldwide [4,5]. Despite extensive therapeutic interventions, it is predicted that this trend will continue, with the annual death projected to increase to about 24 million by 2030 [6,7]. Ischemic heart disease and stroke, both clinical manifestations of atherosclerosis, are the leading cause of death among CVD, accounting for 84.9% of cardiovascular deaths [8]. In addition to ischemic heart diseases, the development of CVD is also greatly influenced by dyslipidemia [9], poor glycemic control, oxidative stress [10], inflammation, obesity, hyperhomocysteinemia, smoking, inactivity, dietary factors [11], and a host of other factors.

Fatty acids (FAs) are crucial structural elements of biological membranes and a source of energy for living things [12]. Additionally, they are vital regulators of a variety of physiological processes, including oxidative stress, inflammation, lipid metabolism, and glycemic control [13]. These physiological processes are intricately associated with the pathogenesis of metabolic syndrome disorders and CVD [14]. Functional lipids are dietary substances that may have an impact on human health by lowering the risk of illnesses and enhancing the quality of life [15]. Numerous functional lipids, including eicosapentaenoic acid (EPA), docosahexaenoic acid (DHA), α-linolenic acid (ALA), linoleic acid (LA), and oleic acid (OA), have been reported to have potential health benefits in the prevention, management, and treatment of CVD and positive effects on cardiometabolic health [15,16], which acts as a boost to the overall health of the individual.

Nonetheless, it is also very important to consider the impact of several other unusual FAs, such as furan, docosapentaenoic acid (DPA), and conjugated FAs, on cardiometabolic health or CVD risks, considering that these FAs are present at low concentrations or absent from typical meals. Most CVDs are caused by risk factors that can be controlled, treated, or modified, such as high blood pressure, cholesterol, diabetes, tobacco use, inactivity, and overweight/obesity. Socioeconomic status and the environment in which an individual lives have a significant impact on exposure to certain CVD risk factors. Considering the available data, it is reasonable to draw conclusions that leading a healthy lifestyle and eating a balanced diet are the best tools for combating CVDs and the best ways to stop them from occurring in society [17]. However, there is still insufficient and contradictory information in the literature about their effectiveness and associated mechanisms of action. Moreover, further research is required to properly elucidate the dosages, dietary intake, effectiveness, and mechanisms of action of these functional lipids and their metabolites. Additionally, studies on the development of valid disease biomarkers and their long-term effects in humans are still scanty. The objective of this review, therefore, is to evaluate available literature regarding these functional lipids and their CVD health benefits.

## 2. Methodology and Data Collection

With a comprehensive search of the PubMed database, published articles on functional lipids and CVD reduction were collected. Additional data searches were performed using other online platforms. Reference citations were performed using EndNote Version 9, making use of trusted sources such as PubMed, NCBI, Web of Science, Science Direct, Google Scholar and other trusted journals. These literature searches were also performed using the keywords “dietary”, “cardiovascular diseases”, “biomarkers”, “supplements”, “eicosanoids”, “polyunsaturated fatty acids”, “chronic diseases”, “health”. Specifically, the main focus was to include recently published clinical studies that will highlight new knowledge on the CVD reduction of functional lipids, especially as reported in the last 10 years, as much as is possible. Firstly, a general overview of CVD was given, followed by the bioactivities of functional lipids, the various mechanisms of action by which CVD reduction is modulated, and finally, an overview of the limitations, challenges, and future perspectives.

## 3. Functional Lipids

**Figure 1:**
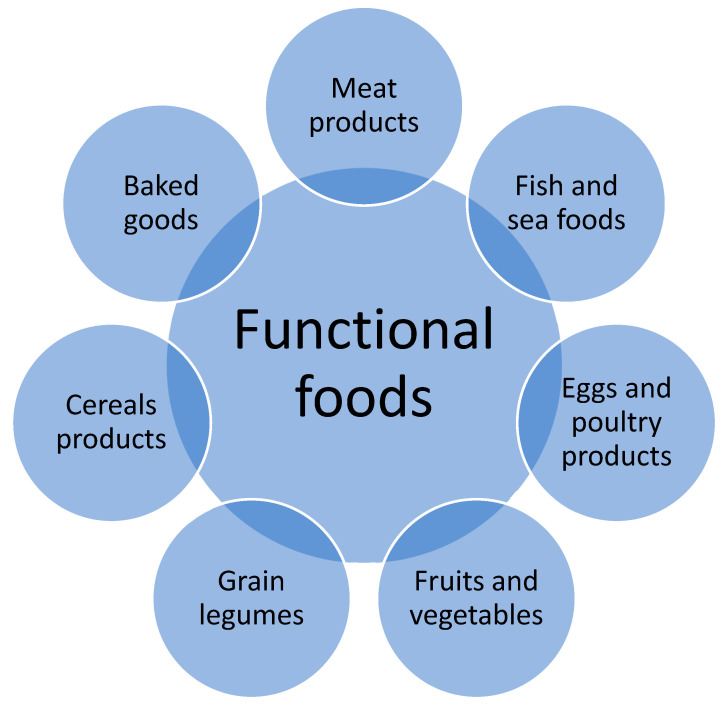
Sources of functional food.

Functional foods are important for human health as they are considered a substantial source of vital nutrients and can be taken as a dietary supplement [15,18,19,20]. Various fruits, vegetables, grains, fish, dairy, and meat products are naturally regarded as the main sources of functional foods (Figure 1). They provide beyond basic nutrition, beneficial physiological hormone-like health effects and/or reduce the risk of developing diseases, including immune-modulatory responses, reduced risk of cancer, osteoporosis, cardiovascular problems, obesity, and many others [20,21,22,23].

Functional lipids include ω-3 (α-linoleic acid, ALA, EPA, and DHA) and ω-6 FAs (gamma linoleic acid (GLA), linoleic acid (LA), conjugated linoleic acid (CLA), medium-chain triglyceride (MCTs) oils, and phytosterols [24,25]. Humans cannot synthesize ω-3 and ω-6 FAs de novo. This is because humans and other animals lack the desaturase enzymes needed to make the most basic members of these families (ALA and LA). Consequently, ALA and LA are regarded as “essential fatty acids” (EFAs) that must be supplied by and/or consumed through the diet. EFAs function as building blocks to produce longer-chain, ω-3, and 6 FAs. In addition to playing numerous more physiological roles, such as reducing inflammation in heart disease, inflammatory bowel disease, lowering cholesterol levels, preventing hardening of the arteries, lowering blood pressure, and neutralizing or lowering levels of inflammatory markers, ω-3 and ω-6 FAs are crucial parts of cell-membrane phospholipids [15,24].

Recently, interest in functional foods has increased dramatically due to the ever-growing self-care movement and industry, evolving food regulations, and a wealth of scientific evidence that emphasizes the critical link between nutrition and health [24]. They are considered a subset of functional foods that resemble traditional foods taken as a part of the regular diet [26,27]. Functional lipids have been shown to have potential physiological benefits and/or reduce the risk of chronic illness in addition to their nutritional functions such as modulating obesity, Alzheimer’s disease, depression, atopic dermatitis, and bone health [15,28]. ALA, EPA, and DHA, omega-3 (ω-3 or *n*-3), and omega-6 (ω-6 or *n*-6) FAs, conjugated linoleic acid (CLA), medium-chain triglyceride oils, and phytosterols are examples of functional lipids [24].

### 3.1. Dietary Sources of Functional Lipids and Recommendations for Their Intake

Table 1 provides a list of dietary sources of functional lipids [25]. The World Health Organization (WHO) recommends adults consume from 0.5–2% of their total calories as ω-3 FAs and from 2.5–9% percent as ω-6 FAs. For adult pregnant and lactating females, the recommended daily intake of EPA + DHA is 0.3 g/day, of which at least 0.2 g/day should be DHA for optimal adult health and fetal and infant development. Based on experimental data, the upper safe intake level for EPA + DHA was determined to be 2 g/day [29]. According to Ras et al. [30], the average dietary intake of phytosterols in European nations ranges from 250 to 400 mg per day with substantial variability, which is comparable to dietary cholesterol intake. The amount consumed may change depending on the predominant dietary pattern; vegan diets have been shown to include the highest amounts (up to 500 mg/day). Sitosterol, which makes up between 60 and 70 percent of all dietary phytosterols, is the most prevalent, followed by campesterol (16 percent) and stigmasterol (10 percent), while sitostanol, campestanol, and 5-avenasterol provide a combined 10 percent [31].

Kojima et al. [32] reported that 6 g of medium-chain triglycerides (MCTs) per day may dramatically enhance a few blood markers of nutritional status. It was also discovered that consuming MCTs in a single dose of 5–10 g effectively increased energy consumption more effectively [33].

Benjamin et al. [34] reported that the majority of CLA research used daily dosages ranging from 3.2–6.4 g, although there have been no reports of any major unfavorable side effects in humans at doses up to 6 g per day, implying their safety at the recommended dose. The FDA grants CLA the GRAS (generally regarded as safe) category and permits its addition to foods. However, it is important to warn that the danger of side effects increases with increasing dosages [35].

#### 3.1.1. Omega-3 Fatty Acids

ω-3 FAs have one of their double bonds, which is three carbon atoms from the methyl end of the molecule [29,36]. ALA is the most quantitatively significant ω-3 FA in the diets. Other essential ω-3 FAs are DPA (20:5*n*-3), EPA (20:5*n*-3), and DHA (22:6*n*-3) [37]. ω-3 FAs have generated increased research interest since claims of their beneficial role in promoting health and lowering the risk of several diseases, including cancer, heart disease, type 2 diabetes, cognitive decline, neurodegenerative diseases, and mental disorders [38,39]. Food sources include nuts like walnuts, seeds like chia, dairy products, eggs, algae, and vegetable oils such as flaxseed, canola, soybean, and hemp oil. It can also be found in the meat of free-range animals, especially herbivores and carnivores [40,41]. Marine oils, rich in ω-3 FAs, have therefore become one of the most popular dietary supplements globally [42].

The main sources of EPA and DHA for the human diet are now marine fatty fish like salmon, mullet, and mackerel. However, alternative sources of EPA and DHA, including bacteria, fungus, plants, and microalgae, are currently being studied for commercial production [43]. Plants need arable land, have longer growth durations, and lack the enzyme activity necessary to produce the long-chain PUFAs, EPA and DHA, unless they are genetically engineered. Fungi, on the other hand, require an organic carbon supply and often have extended growth periods [44]. Microalgae are the first producers of EPA and DHA in the marine food chain. They have a great potential for producing long-chain ω-3 FAs and can grow quickly in a variety of autotrophic, mixotrophic, and heterotrophic culture conditions [45]. PUFAs are essential to human metabolism since they are involved in numerous physiological and metabolic functions. Additionally, they have a significant structural role in cell membranes and support several membrane functions, including signal transduction, permeability, fluidity, and the activity of enzymes and receptors that are bound to membranes [29].

According to Sokoła-Wysoczańska et al. [37], ALA acts as a precursor to other ω-3 FAs. ALA can be transformed by the body into EPA and DHA, which means that it can regulate the physiological activity of these FAs even if they can also be obtained from the food. EPA and DHA are usually referred to as “marine ω-3” since they are commonly found in fish and seafood, particularly in fatty fish oils, squid and krill oil, egg oil, and seaweed [46]. A series of desaturases and elongases sequentially convert ALA to EPA, DPA, and, to a lesser extent, DHA in animal tissues, although this conversion process is not applicable in plant tissues [39,47].

#### 3.1.2. Alpha-Linolenic Acid (α-Linoleic Acid, ALA)

ALA (C18:3) belongs to the family of essential ω-3 PUFA and contains three double bonds at positions 9, 12, and 15. ALA is an essential FA that is mostly present in plant oils like flaxseed and rapeseed oils and is a crucial component of a mixed diet [48]. According to the results of a recent Cochrane meta-analysis that included 79 randomized controlled trials, raising ALA may marginally lower the risk of CVD events and probably slightly lower the risk of ischemic heart disease (IHD) mortality and arrhythmia [12]. A similar outcome was obtained in a meta-analysis conducted by Pan et al. (2012), which reported that there may be potential cardiovascular benefits from ALA consumption. Specifically, a 10% reduction in the risk of death from IHD is linked to an increase in ALA intake of 1 g/day [49].

Bloedon et al. [50] reported that increased flaxseed oil consumption can, in the short term, lower LDL-cholesterol, lipoprotein(s), and HDL in a double-blind, randomized controlled clinical trial. In a randomized controlled study, walnuts and fatty fish were given as dietary supplements, and the results showed that ALA had a suppressive effect on blood levels of total cholesterol, LDL, and TG concentrations [51]. There are significant inverse correlations between the blood concentration of both ALA and DHA and the intima-media thickness of the internal carotid artery [52]. The average carotid artery intima-media thickness was adversely correlated with ALA in a small crossover trial involving individuals who had experienced their first myocardial infarction (MI) [53]. Lemaitre et al. [54] reported that an increase in ALA levels is associated with an increased risk of sudden cardiac arrest. Although more prospective research is required to fully understand the link between ALA and circulatory system disorders, there is a common scientific agreement that ALA provides modest cardiovascular protective benefits [55].

#### 3.1.3. Eicosapentaenoic Acid (EPA) and Docosahexaenoic Acid (DHA)

Essential PUFA, EPA (C20:5) is one of the primary constituents of complex lipids. The mechanisms underlying the effect of EPA on the development of atherosclerosis include effects on oxidative stress, endothelial dysfunction, and increased synthesis of eicosanoids, which is associated with dilation of blood vessels and reduction of inflammation and thrombogenesis, alleviation of atherogenic dyslipoproteinemia, and other effects [15,56]. EPA given at a dosage of 1.8 g/day was found to reduce cardiovascular events in statin-treated patients by 19% and to lower blood concentrations of LDL by 25% in the large prospective randomized clinical trial [57].

Patients who received EPA also experienced a considerable reduction in unstable angina pectoris and coronary manifestations. It was concluded that EPA is a promising functional FA for patients with HC in the prevention of significant coronary events, particularly nonfatal ones. The Reduction of Cardiovascular Events with Icosapent Ethyl-Intervention Trial (REDUCE-IT) provides the best data to date regarding the effect of ω-3 FAs in reducing the incidence of atherosclerotic cardiovascular disease [58]. The REDUCE-IT results with icosapent, a stable and very pure EPA ethyl ester, showed a significant reduction in the risk of major ischemic events, including cardiovascular death, in individuals with excessive triglyceride levels who received 2 g of icosapent twice daily.

The benefits of EPA for endothelial function include improving the ratio of nitric oxide to peroxynitrite in human umbilical vein endothelial cells (HUVECs) and acting synergistically with statins [59]. EPA reduces reactive oxygen species (ROS) production, adhesion molecule and cytokine expressions, activation of apoptosis-related proteins, and HUVEC apoptosis caused by palmitic acid [60,61]. Furthermore, EPA prevents membrane vesicles’ lipid peroxidation processes [62]. EPA’s ability to scavenge and quench ROS generation and maintain the structural integrity of lipid membranes may be the cause of these antioxidant qualities [59,62]. Additionally, EPA is integrated into the lipid bilayer. EPA has been reported to prevent ischemia damage by causing neovascularization involving human endothelial-cell progenitors [63]. Due to its high lipophilicity, EPA may have anti-inflammatory and antioxidant properties, lessen the adherence of monocytes to the endothelium, prevent the buildup of macrophages and foam cells in lipid spots, and thicken the fibrotic layer that covers lipid-rich plaque [56,64,65].

The proportion of EPA in the phospholipids of stable plaques is inversely correlated with the plaques’ level of inflammation and T-cell density [66]. Despite the main disorders being treated, EPA reduces the intima-media thickness of the carotid artery in patients with hypertriglyceridemia and patients with atherosclerosis risk factors [67,68]. EPA is also involved in synthesizing resolvins and unique proteins that have the potential to modulate inflammatory processes [69,70]. Both substances reduce blood-derived neutrophil recruitment, which helps to alleviate inflammatory blood vessel processes associated with atherosclerosis [71]. EPA may lessen platelet aggregation in addition to limiting the size of the adjacent clot by reducing platelet aggregation and thereby minimizing MI volume, involving the rupture of an atherosclerotic plaque, which can cause acute coronary syndrome [56]. Furthermore, EPA helps to reduce blood inflammatory biomarker (cytokines, C-reactive protein, CRP) levels and enhances blood lipid profiles, which prevents the development of clinically significant cardiovascular events. Using EPA as an adjuvant therapy reduces the risk of CVD through various mechanisms, including triglyceride (TG) lowering, membrane stabilization, and antithrombotic, anti-inflammatory, or antiarrhythmic properties [72,73].

DHA (C22:6) has a distinct stereochemical structure, the highest level of unsaturation, and it prevents heart and blood vessel spasms by ensuring efficient signal conductance in neurons [74]. It also has the potential to have antithrombotic, antiatherogenic, antiarrhythmic, and vasoprotective effects. DHA decreases blood TG levels by lowering liver enzyme activity such as alanine aminotransferase (ALT) and/or aspartate aminotransferase (AST) [75].

Concurrently, there was a rise in blood HDL levels due to increased phospholipid production. DHA has been shown to reduce inflammatory indicators such interleukin-1β, tumor necrosis factor α (TNF-α), and interleukin-6 [76,77]. In patients with IHD, the blood level of DHA is related to endothelial function, suggesting that endothelial dysfunction may be marked by a low DHA content [78]. Also, there is evidence that DHA can mitigate the abnormalities associated with endothelial function occasioned by a high-fat diet [79]. However, the specific ω-3 PUFA that has the biggest impact on blood lipid profile is a subject of extensive review [80]. While EPA and DHA have similar effects on TG levels, they have distinct effects on HDL and LDL cholesterol levels [81,82]. In addition, the prevalence of CVD is inversely correlated with circulating DHA and EPA concentrations [80].

#### 3.1.4. Omega-6 Fatty Acids

Omega-6 FAs, like ω-3 FAs, are PUFAs that may be found in vegetable oils, nuts, and seeds. Linoleic acid is the primary dietary ω-6 FA [24]. When ingested, linoleic acid can undergo chain elongation and desaturation to become longer-chain FAs like arachidonic acid [48]. Two main functions of ω-6 FAs in the body are as membrane structural components that modulate membrane function and as precursors of eicosanoids that control inflammatory responses, renal and pulmonary function, and vascular tone [83].

In comparison to ω-3 FAs, these PUFAs have received less attention. However, most studies suggest that PUFAs are generally beneficial in preventing CVD risk factors such as dyslipoproteinemia, hypertension, and atherosclerosis [84]. Increased inflammation from high ω-6 PUFA compared to ω-3 PUFA levels is thought to exacerbate cardiovascular risk [85]. Consequently, there is ongoing discussion on the pro- or anti-inflammatory nature of ω-6 PUFA’s actions [86]. There are contradictory opinions regarding the roles of these PUFAs, as some studies have reported negative outcomes of PUFAs, especially with ω-6 PUFAs having pro-inflammatory qualities, while others reported that ω-3 FAs appear to have cardioprotective effects [87,88]. However, there is currently little or a lack of evidence from human studies to support this notion. These effects were assessed for at least 12 months in a recent Cochrane meta-analysis that compared higher and lower intakes of ω-6 FA in people with or without CVD [48]. The study included 19 randomized controlled trials and 6461 participants [89]. The authors discovered that raising ω-6 PUFAs could lower myocardial infarction (MI) risk. Increasing ω-6 PUFA intake may help those who are at high risk of MI, even though its advantages are still unknown [90].

Over a minimum of one year, it has been demonstrated that elevating ω-6 PUFA lowers serum total cholesterol levels but not other blood lipid fractions. Furthermore, the intricate biochemistry of eicosanoids, along with docosanoids and octadecanoids, has been more apparent in recent times, suggesting that the ω-6 PUFA class itself can no longer be regarded as pro-inflammatory [90]. These new findings began to erode the view that PUFAs could be encapsulated in a single, straightforward ω-6/ω-6 ratio. Changes in the participation of ω-3 and ω-6 FAs in the daily nutritional ratio are vital for ensuring the body has the right amount of ω-3 FA reactions and the possibility of good health [48].

#### 3.1.5. Gamma Linoleic Acid (GLA)

GLA (C18:3) is a PUFA consisting of a chain with 18 carbon atoms and three double bonds at positions 6, 9, and 12. Although it can be found in oils made from different seeds, GLA is often used as a dietary supplement [91]. A possible indication for GLA nutritional supplementation is a confirmed lower GLA content of phospholipids and cholesterol esters in the blood of patients with hypertriglyceridemia or HC [92]. The GLA can also exert anti-inflammatory and anti-proliferative effects, as well as lower lipid levels [93]. There is limited data to support the benefit of GLA supplementation on cardiovascular events and CVD mortality rates, but in research by Schwab et al. [94], blood concentrations of TG, TC, and LDL were considerably decreased while the HDL fraction was upregulated. GLA has also been reported to protect blood vessels by lowering arterial hypertension and, via the blood-clotting system, aids in reducing complications from IHD [95].

#### 3.1.6. Conjugated Linoleic Acid (CLA)

Positional and geometric isomers of PUFAs containing conjugated double bonds are collectively referred to as conjugated FAs [96]. CLAs like cis-9, trans-11, and trans-10 CLA, as well as conjugated linolenic acids (CLNAs) such α-eleostearic acid, punicic acid, and jacaric acid, are the most commonly occurring conjugated FAs. The primary naturally occurring isomer of CLA, cis-9, trans-11 CLA, was initially discovered to be a transitional form used by rumen bacteria to convert PUFAs into saturated stearic acid. This explains its presence in ruminant-animal-derived foods such as dairy products and meat from sheep, goats, and cattle [96,97].

Plant seeds, including tung, bitter gourd, snake gourd, and pomegranate seeds, as well as trichosanthes, pot marigold, jacaranda, and catalpa seeds, naturally contain CLNA [98]. CLNA may convert into CLA in vivo, according to several studies conducted in both humans and animals [99].

Studies on humans and animals have shown that CLA has few significant impacts on CVD and its risk factors. A randomized control trial (RCT) found that supplementing with cis-9, trans-11 CLA for six months had no significant effect on blood pressure, insulin resistance, glucose, lipids, body composition, or the 10-year absolute risk of fatal cardiovascular disease (CVD) as determined by the European Systematic Coronary Risk Evaluation (SCORE) formula in subjects who were overweight or obese [100]. Results on the risk of CVD have been consistent [101,102].

Lipid peroxidation and CRP were observed to be considerably elevated in one RCT with supplementation of trans-10, cis-12 CLA, another isomer of CLA [103]. CLA (a blend of cis-9, trans-11 CLA and trans-10, cis-12 CLA, 50:50) was found to significantly lower CRP in atherosclerotic patients in just one RCT [102]. Other studies on animals also showed that CLA (cis-9, trans-11 CLA, trans-10, cis-12 CLA, or their mixture) protected against atherosclerosis [104,105]. By targeting β2 integrin expression, CLA could inhibit monocyte adhesion in vitro [106]. This could be another mechanism for the anti-atherosclerotic effect of CLA. In mammary epithelial cells treated with lipopolysaccharide (LPS), CLA (combination of cis-9, trans-11 CLA, and trans-10, cis-12 CLA) reduced the expression of pro-inflammatory cytokines (including IL-1β, IL-6, and TNFα) by suppressing the generation of reactive oxygen species (ROS) and increasing the expression of PPARγ [107].

### 3.2. Medium Chain Triglycerides (MCTs)

Medium-chain FAs, MCFAs, make up medium-chain triglycerides, MCTs [108]. These saturated FAs, which have a chain length of 6–10 carbons, are called mixed triacylglycerols. They include hexanoic acid (C6:0, also known as capronic acid), octanoic acid (C8:0, also known as caprylic acid), decanoic acid (C10:0, also known as capric acid), and dodecanoic acid (C12:0, also known as lauric acid). They include medium-chain FAs at all three of the glycerol backbone’s locations [109,110]. Medium-chain triglycerides can be found in small amounts in natural sources, such as coconut oil, palm kernel oil, and bovine milk. In the 1950s, MCTs were developed as byproducts of the production of coconut oil, and studies into their potential uses were launched. They have now found extensive use in both food and non-food applications [108,111]. Despite being categorized as saturated FAs, MCFAs have different physiological, physicochemical, and nutritional properties from so-called long-chain saturated FAs [112,113].

MCFA and glycerol undergo an esterification reaction to produce synthetic MCTs [114,115]. The MCT has a shorter chain length and provides 8.4 kcal/g of energy at room temperature, making it less caloric than long-chain saturated triglycerides [109]. They were introduced as a remarkable energy source for a range of clinical nutrition needs, including severe hyperchylomicronemia, atherosclerosis, obesity, parenteral nutrition, and malabsorption of fat. They were also utilized in formulations for infants [116,117,118].

#### 3.2.1. Phytosterols (PS)

Most plant cells contain phytosterols, fat-soluble members of the triterpene family that contribute to the stability and structure of membranes. According to Moreau et al. [119], they have a tetracyclic structure with a side chain at position 17 of the D ring. They have a comparable structural role as cholesterol, which is by far the most abundant sterol in animal cells. Their structures are quite similar. When it comes to side chain binding at position *C*-17, phytosterols are different from cholesterol. For instance, sitosterol has an ethyl group connected in *C*-24 of the side chain, whereas campesterol has a methyl group in the same place that is empty in cholesterol [120]. Five alpha-saturated phytosterol derivatives are known as phytostanols [121]. Numerous phytosterol molecules have been found in plant cells, with beta-sitosterol, campesterol, stigmasterol, brassicasterol, and avenasterol being the most prevalent types [122,123].

Oily fruit, oil seeds, and the oils derived from them have the highest food content in phytosterols [124]. The oils with the highest concentration of phytosterols are rapeseed oil, wheat germ oil, and corn oil; pistachios have the highest concentration of all the oily fruit varieties [125]. Legumes and grains also contain phytosterols, but fruits and vegetables have considerably less amounts. According to Wang et al. [126], there are significant variations in the total phytosterol content of vegetables, ranging from a few milligrams or tens of milligrams per 100 g of fruit and vegetables to over 1000 mg per 100 g in some vegetable oils.

The total daily dietary intake of phytosterols in European nations ranges from 250 to 400 mg, with significant variation [30]. This figure is comparable to the dietary cholesterol intake. Depending on the prevailing dietary pattern, the amount consumed may change; vegan diets have been shown to include the highest amount (up to 500 mg/day). Sitosterol makes up roughly 60–70% of all dietary phytosterols, with campesterol (16%) and stigmasterol (10%) following closely behind. Together, sitostanol, campestanol, and ∆5-avenasterol comprise less than 10% [31].

Phytosterols reduce the absorption of cholesterol, which lowers blood levels of LDL and HDL and lowers the risk of cardiovascular disease [122]. Plant sterols have a higher affinity for fat-digesting micelles and are more hydrophobic than cholesterol. Consequently, they can displace intestinal cholesterol from the micelles, thereby reducing the absorption of intestinal cholesterol [127]. Numerous studies have demonstrated that PS supplementation lowers both total cholesterol (TC) and LDL-cholesterol (LDL-C). Although there was much debate in the past over the relative efficacy of food-based versus capsule PS carriers, current clinical trials have demonstrated that oral PS supplements, both tablets and capsules, had a comparable LDL-C lowering effect on fortified meals [30,128,129]. Nonetheless, there is a decrease in the incorporation of cholesterol into micelles when PS is present. It is widely believed that the inherent hydrophobic structural properties of PS, which compete with and displace cholesterol and increase fecal loss, are the causes of decreased cholesterol micellization [127].

#### 3.2.2. Omega-6 to Omega-3 Ratio of the Diet

For decades, PUFAs have piqued the interest of scientists all around the world [130]. Strong and consistent data supported the benefits of PUFA for cardio protection and hypercholesterolemia [131,132]. A lower risk of major chronic diseases, such as diabetes, Alzheimer’s disease, and CVD, has been associated with the consumption of ω-3 PUFA, particularly marine long-chain PUFA (EPA), and DHA) [36,116,133].

Nonetheless, there is still conflicting data about the relationship between mortality and ω-3 PUFA consumption. However, certain observational studies [77,134,135], found a correlation between reduced overall mortality and greater circulating levels or dietary intake of ω-3 PUFA. Additionally, conflicting results regarding the effects of ALA and marine ω-3 PUFAs on mortality have been documented [136,137]. Up until now, there has been disagreement over the possible dose–response relationship and health effects of ω-6 PUFAs. Concerns regarding the potential pro-inflammatory and pro-thrombotic effects of ω-6 PUFAs have also been raised by the public [84].

Nevertheless, this pro-inflammatory impact has not yet been validated by high-quality evidence from human studies [90]. However, some studies reported correlations between the rising ω-6/ω-3 ratio and the prevalence of obesity, cancer, and CVD [138,139]. Unfortunately, not much research has been carried out to shed light on how ω-6 PUFA and mortality are related. Consequently, there is not yet enough evidence for a probe about this association. Presently, there is also a dearth of information regarding PUFA intake and mortality in the Chinese population [140,141]. Using two nationally representative cohorts in China and the U.S. National Health and Nutrition Examination Survey (NHANES), the relationships between PUFA intake and both total and cause-specific death were thoroughly evaluated [140,141]. It is suggested that the human diet should have a healthy ratio of ω-6 to ω-3 FAs, which falls between 1:1 and 4:1 [142]. This ratio is presently greater in most Western diets because of the decreased consumption of fish and the overuse of vegetable oil, which is rich in LA, in the human food chain [40]. To convert to arachidonic acid (AA) and EPA, respectively, LA and ALA compete with one another for the same enzymes. Therefore, the availability of the physiologically active compounds, AA and EPA, will be determined in part by the ratio between LA and ALA. The ratio of AA to EPA may therefore affect immunological responses, smooth muscle contraction, blood coagulation, and inflammation since AA and EPA compete with one another for the same enzymes needed for eicosanoid production [143].

Redness, swelling, heat, and discomfort are important responses that are caused by the ω-6 fat linoleic acid and the ARA it generates. Conversely, resolvins generated from long-chain ω-3 FAs, EPA, and DHA are meant to quickly diminish acute inflammatory reactions. The prevention of an excessive and prolonged inflammatory response, which may result in tissue damage and may be an autoimmune illness, may therefore depend on maintaining a healthy ω-6/3 ratio in the diet. High ω-6/3 ratios are associated with prolonged low-grade inflammation and predispose to supraphysiologic inflammatory responses. It has been suggested that a lack of EPA and DHA and excessive linoleic acid consumption, primarily from synthetic ω-6 seed oils, contribute to the population’s pro-thrombotic and pro-inflammatory tendencies.

High ω-6 FA consumption and a high ω-6/3 ratio have also been associated with weight increase in both human and animal studies, while high ω-3 FA intake lowers the risk of weight gain. Lowering the LA/ALA ratio in animals results in a decrease in overweight and obesity. The n ω-6/3 ratio is increased by the ingestion of plant oils high in *n*-6 PUFA and relatively low amounts of marine foods, which are high in *n*-3 PUFA. Less competition for Δ6 desaturase results in higher amounts of EPA and DHA in muscle tissue when one eats a diet high in ALA and low in LA [84].

It is imperative to try and limit the consumption of ω-6 FAs while increasing the consumption of ω-3 FAs. Two possible approaches would be firstly, to replace dietary vegetable oils high in ω-6 FAs (corn, sunflower, safflower, cottonseed, and soybean oils) with ω-3 rich oils (flax, perilla, chia, and rapeseed) and monounsaturated oils (olive, hazelnut, or the new high monounsaturated sunflower oil); and secondly, to increase fish consumption to 2–3 times a week while reducing meat consumption [138]. According to Kłosiewicz et al., the Polish Forum of Cardiovascular Disease Prophylactic Program recommends a 4:1 ω-6/3 ratio. Nevertheless, a ratio of 10:1 or greater is becoming increasingly common in literature [84]. The human body may be negatively impacted by both PUFA scarcity and overuse. It is therefore reasonable to predict that environments with high ω-6/3 ratios will exhibit a higher inflammatory condition, although the issue is not so much a high content of ω-6 PUFA as it is an absence of ω-3 PUFA [48].

## 4. Roles of Functional Lipids on Health

Foods often contain fats and lipids, which may have vital functions. In terms of health and disease, their quality might matter more than their quantity. Functional lipids have been connected to the treatment and prevention of numerous diseases by recent studies [15] including CVD (Table 2). The functional lipids, which include medium-chain triglycerides, CLA, ω-3 and 6 FAs, and phytosterols, are numerous and include treatment and management of blood pressure, cardiovascular health, diabetes, obesity, and bone health.

## 5. Cardiovascular Disease (CVD) Reduction

The leading cause of death worldwide is CVD [151]. The risk of CVD is strongly correlated with several risk factors, including smoking, dyslipidemia, hypertension, obesity, a sedentary lifestyle, and ethnicity [151]. High serum concentrations of triglycerides, low-density lipoproteins, very-low-density lipoproteins, total cholesterol, and low levels of high-density lipoproteins are risk factors for developing CVD [152]. A suggested dietary approach to reduce risk factors is to substitute mono- and PUFAs for some of the dietary saturated FAs [153,154,155,156]. It is noteworthy that behavioral risk factors may be able to prevent up to 90% of CVD cases [157]. This suggests that nutrition and dietary variables have a close relationship with CVD. However, some questions have been raised about this recommendation [158,159,160]. The advice to consume LA, an important *n*-6 PUFA, is one such topic of debate [161,162,163]. For instance, it has been proposed that substituting LA for saturated fat lowers serum cholesterol but has no effect on the risk of coronary heart disease (CHD)-related mortality [159]. Furthermore, there has been concern that a high-LA diet may raise the risk of inflammation [164].

Higher tissue and circulation concentrations of LA were substantially linked to a lower risk of cardiovascular events in a recent study of 30 prospective observational studies [148]. Higher levels of circulating LA (but not other *n*-6 PUFAs) reduced overall and CHD mortality in older persons, according to the Cardiovascular Health trial, a prospective cohort trial [165]. Total dietary PUFAs, including LA and ω-3 PUFAs, were found to be inversely correlated with CVD mortality in one population-based cohort study [166]. Furthermore, low *n*-6 PUFA consumption and larger intakes of saturated and trans fats increased CHD mortality, per meta-analyses of prospective cohort studies [77].

PUFAs have been reported to exhibit inconsistent effects on atrial fibrillation (AF) depending on the type of PUFA as well as the quantity and dose. With *n*-3 PUFA, the risk of AF may be increased, especially at higher doses compared to placebo, in a way not seen with other studies in which the risk of AF after coronary artery bypass surgery was reduced [145]. However, with *n*-6 PUFAs, especially LA, the risk of AF is lowered, especially in men without a history of CHF or CHD [49,148].

ω-3 FAs have been shown to significantly reduce the incidence of sudden mortality from cardiac arrhythmias in those with pre-existing coronary heart disease [167]. Eating ω-3 FAs from plants and seaweed is beneficial for people at risk of CHD, according to extensive epidemiologic studies. While the ideal amount is unknown, results from prospective secondary prevention studies indicate that 0.5 to 1.8 g of EPA+DHA per day (from fatty fish or supplements) is quite beneficial [84]. According to studies by Tavazzi et al. [168] and Marchioli et al. [169], EPA+DHA has been associated with a decreased risk of heart failure events, recurrent coronary artery events, and sudden cardiac death after an acute myocardial infarction. The administration of 1800 mg of EPA per day resulted in a significant reduction in CIMT (carotid intimal-medial thickness) and an improvement in brachial-ankle pulse wave velocity in patients with type 2 diabetes, suggesting a decrease in atherosclerosis and an improvement in endothelial function [170].

According to another study by Venty et al. [171], administering virgin coconut oil (VCO) to white Wistar rats on a high-cholesterol diet raised HDL levels while lowering LDL and TC levels. This was because the MCT content in VCO suppressed lipogenesis. The administration of VCO, which contains 60% MCT, can significantly increase HDL with no change in LDL or in individuals with coronary artery disease [172]. Randomized controlled trial (Twelve Weeks, in 10 lipid-normal women) who did not receive coconut oil supplementation (COS) in conjunction with physical activity and 10 women with normal lipid profiles received coconut oil supplementation (COS) in conjunction with exercise. There was a 3% drop in LDL in the COS group [80].

Numerous studies have shown that consuming 2 g of phytosterols per day can significantly lower LDL-C (8–10%) [121]. Data, however, do not support the idea that consuming phytosterols lowers the incidence of CVD. Conversely, the concurrent rise in phytosterol plasma concentration may raise the risk of atherosclerosis development [173,174]. After four weeks, there was a 10.3% drop in LDL-C levels, according to a study by Vásquez-Trespalacios and Romero-Palacio [175] that assessed the use of 4 g/day of plant stanols. Gylling et al. [176] demonstrated a 17.1% decrease in LDL-C levels in their trial, which used the maximum dose of phytosterols (8.8 g/day of plant stanols). One hundred and twenty-four studies with a mean phytosterol dosage of 2.1 g/day (range 0.2 to 9.0 g/day) were included in a meta-analysis by Ras et al. [150]. A 6 to 12% reduction in LDL-C content was linked to a daily intake of 0.6 to 3.3 g.

### Mechanism of Cardioprotection

**Figure 2:**
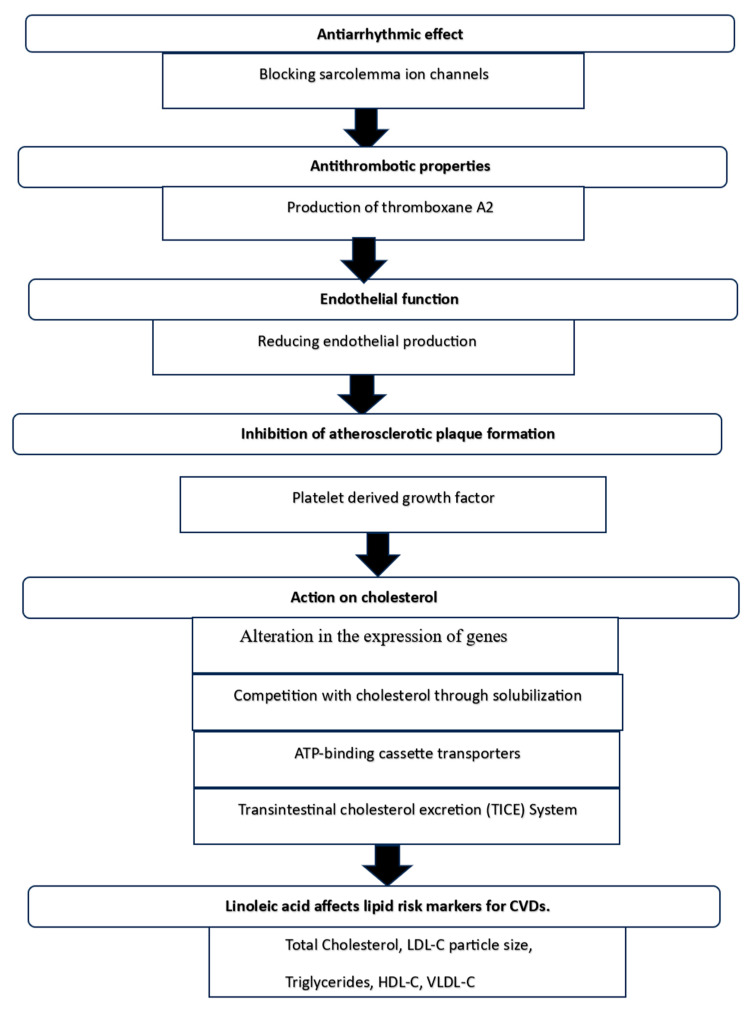
Suggested mechanisms for the modulation of CVD by functional lipids.

Numerous mechanisms have been demonstrated for the cardioprotective properties of functional lipids, including antiarrhythmic, antithrombotic, endothelial function, inhibition of atherosclerotic plaque formation, cholesterol lowering, and several others as discussed in the following section (Figure 2). According to several studies [167,177,178], ω-3 FAs are hypothesized to maintain the electrical activity of cardiac myocytes by blocking sarcolemmal ion channels. This prolongs the relative refractory period.

The thrombotic effects of ω-3 FAs are noteworthy. According to Piper and Garelnabi [179] and Shahidi et al. [38], EPA has been demonstrated to prevent the production of thromboxane A2, a prostaglandin that induces platelet aggregation and vasoconstriction. Consuming EPA has also been demonstrated to lower platelet reactivity and adhesion, which shows up as longer bleeding times and fewer platelets adhering to glass beads [180]. Other antithrombotic effects that have been documented include decreases in fibrinogen and elevations in tissue plasminogen activator [181].

Since EPA increases the vasodilatory impact of nitrous oxide, ω-3 FAs also positively affect endothelial function [181]. It has been demonstrated that administering fish oil to people reduces neutrophil generation of oxygen-derived free radicals. It has been proposed that the bioavailability of nitrous oxide is increased by this decrease in free radicals. Research employing ultrasonic monitoring of brachial artery flow-mediated vasodilation has revealed enhanced large artery endothelium-dependent dilatation in individuals receiving fish oil therapy [182]. Reducing endothelial production of vascular cell adhesion molecules can also enhance endothelial function by lowering leukocyte adherence to the endothelium [183].

Studies on animals have also demonstrated that EPA and DHA ingestion inhibits the formation of atherosclerotic plaque [167]. Smooth muscle cells and macrophages are two key cells involved in the establishment of an atherosclerotic plaque [184]. A crucial chemoattractant and mitogen for smooth muscle cells and macrophages is platelet-derived growth factor. ω-3 FA consumption reduces messenger RNA synthesis and platelet-derived growth factor production [185].

According to Cohn et al. [186], phytosterols primarily reduce intestinal cholesterol absorption by 30 to 50%, which lowers LDL-C levels. Several mechanisms, including competition with cholesterol through solubilization in mixed micelles in the intestinal lumen, may be responsible for this reduction in the quantity of cholesterol accessible for absorption [187]. Additional mechanisms that have been proposed are: firstly, alteration in the expression of genes that encode sterol-carrying proteins, like the Niemann-Pick C1-like 1 (NPC1-L1) protein, which reduces the amount of cholesterol transported to the enterocyte; secondly, ATP-binding cassette transporters (ABCG5 and ABCG8), which promote the efflux of cholesterol from the enterocytes to the intestinal lumen; thirdly, decreased rate of cholesterol esterification in the enterocyte; and fourthly, increased removal of cholesterol from the body through the transintestinal cholesterol excretion (TICE) system [121].

It has been shown that PUFAs, or linoleic acid, influence lipid risk indicators for CVD. The mechanisms associated with alterations of these risk markers are discussed in the following section. Several studies [84,188] showed that LA reduced total serum cholesterol when compared to other dietary patterns that were not high in PUFAs. It was shown that PUFAs stimulate the transcription of the liver X receptor alpha (LXRα) gene [189], possibly through the action of peroxisome proliferator activated receptors (PPARs). As a result, PUFAs aid in the catabolism of cholesterol by promoting CYP7 activity. LXRα enhances the production of cholesterol 7α-hydroxylase (CYP7), which converts cholesterol to bile acids [84]. It was suggested that PUFA (or LA) consumption lowers total serum cholesterol.

Compared to SFAs, PUFAs have a stronger interaction with PPARα [152]. According to Rakshandehroo et al. [190], PPARα binds itself to peroxisome proliferator response elements (PPREs) found in the promotor regions of genes like lipoprotein lipase (LPL) and apoC-III. It has been suggested that LPL may exhibit enhanced activity towards PUFAs containing VLDL triglycerides, which could result in an increased breakdown of lipoproteins rich in triglycerides, such as chylomicrons and VLDL particles [60]. ApoC-III inhibits LPL activity, which raises triglyceride levels [167]. According to a study by Gugliucci [191], PUFAs have been shown to lower apoC-III, which in turn increases LPL activity and, in fact, VLDL degradation. Additionally, it has been demonstrated that ω-3 PUFAs decrease triglycerides via inhibiting FA synthase, diacylglycerol acyltransferase, and acetyl coenzyme A (CoA) carboxylase [36,192,193]

A study by Bergeron et al. [194] found that consuming a diet high in PUFAs reduces large, buoyant LDL particles, whereas eating a diet low in SFAs enhances the former [195,196]. SFAs are known to raise the hepatic lipase and LPL activity [152,167,197]. Thus, hepatic lipase may promote the breakdown of triglyceride-rich lipoprotein remnants, while LPL promotes large, buoyant LDL particles [158]. To better understand the mechanisms by which specific FAs influence LDL particle size, more study is necessary in this field [197].

In the liver, lipogenesis and cholesterol synthesis are linked to the sterol regulatory element-binding protein-1 (SREBP-1) [198]. PUFAs have been reported to suppress the transcription of the SREBP-1 gene and/or its protein, which lowers the liver’s release of VLDL [195]. Furthermore, PUFA consumption raises VLDL absorption and catabolism [188,193]. Through several processes, including the elimination of cholesterol from macrophages, enhancement of endothelial function, and an increase in antioxidant and anti-inflammatory properties, HDL particles contribute to a lower risk of CVD [199]. Consuming a high-fat, high-cholesterol Western diet resulted in an increase in oxidized lipids in HDL, including oxidized LA and AA [200,201]. It has been proposed that lipoproteins containing oxidized FAs promote atherogenesis [202]. The total cholesterol/HDL-C ratio decreases when SFAs are replaced with MUFAs and/or PUFAs, although LDL-C and total cholesterol concentrations are also slightly lower [153]. To explain the processes by which FAs affect HDL-C, more study is necessary.

## 6. Side Effects of Functional Lipids

### 6.1. Omega-3 Fatty Acids

Evidence from epidemiological, clinical, and experimental investigations suggests that ω-3 PUFAs lower the incidence of certain types of cancer [38]. Multiple clinical studies corroborate these reported outcomes. However, the amount, source, type, and form of ω-3 PUFAs (ethyl esters or triacylglycerols) as well as the ratio of ω-6/3 PUFAs, the proportions of EPA, DHA, and docosapentaenoic acid in the preparations, and genetic factors are some of the factors that have led to inconsistent results regarding ω-3 PUFAs and cancer [203]. Prostate cancer is one of the diseases that appears to be associated with inflammation in carcinogenesis [204,205]. Because of their anti-inflammatory properties, ω-3 PUFAs may have anticarcinogenic benefits [206].

However, the idea that ω-3 FAs lower the risk was not supported by a sizable prospective study that looked at the connection between prostate cancer risk and inflammation-associated phospholipid FAs [207]. According to the study, DHA may raise the risk of high-grade prostate cancer [207]. In a case-controlled study, the highest quintile of plasma phospholipids, EPA and DPA, showed a 14% and 16% increase in prostate cancer risk, respectively, compared to the lowest quintile [208]. The consistency of the findings implies that ω-3 FAs may be a factor in prostate carcinogenesis, even though the correlations do not offer clear proof that consuming fish or fish oil supplements causes prostate cancer [39].

A fishy aftertaste and gastrointestinal disturbances are two further possible adverse effects of ω-3 FAs, and they both seem to be dose-dependent [209]. Certain fish species, such as sharks, swordfish, king mackerel, and tilefish (golden bass or golden snapper), may have a significant concentration of methylmercury, polychlorinated biphenyls, dioxins, and other environmental toxins. The Environmental Protection Agency (EPA) and the Food and Drug Administration (FDA) of the United States issued a statement advising women who are pregnant, women who are not yet pregnant, nursing mothers, and young children to eat fish and shellfish that is lower in mercury and to avoid eating certain types of fish.

A recent survey found that farmed salmon had noticeably greater concentrations of organochlorine contaminants, including polychlorinated biphenyls, than wild salmon. Nevertheless, scientists cannot agree on how many farmed salmon are safe to consume. Most high-quality fish oil supplements are free of these impurities [158]. Most of the research indicates that fish oil does not significantly raise hemoglobin A1C or glucose levels, despite conflicting data regarding its impact on glucose control [109].

### 6.2. Omega-6 Fatty Acids

An imbalance in the ratio of ω-6 to ω-3 FAs can exacerbate issues such as inflammation because a higher concentration of ω-6 FAs generates eicosanoids, which are linked to inflammatory illnesses and the development of thrombus and atheromas [143]. Increased adiposity in the offspring is linked to high perinatal ω-6 FA intake [138]. In human research, the amount of arachidonic acid in adipose tissue is linked to children’s BMI and overweight status. There is a correlation between high subscapular skin-fold thickness at three years of age and high ω-6/3 FAs in the phospholipids of the umbilical cord red blood cell (RBC) membrane [210]. In both humans and rats, high-fat diets high in ω-6 FAs have been demonstrated to increase the risk of leptin resistance, diabetes, and obesity [211,212].

### 6.3. Conjugated Linoleic Acids

There are a few reports of potential side effects, mostly in rats and connected to the 10-trans and 12-cis isomers of CLA, despite the beneficial effects of supplementing with the oil on certain health-related measures. Pro-carcinogenic effects and enhanced prostaglandin synthesis in animal models have been linked to CLA 10-trans and 12-cis [213]. In addition to decreased leptin and an increased risk of developing insulin resistance, further adverse effects could result from an increase in lipid oxidation products, or isoprostanes [214].

Research also indicates that there may be a detrimental change in the serum lipid profile in humans, as seen by elevated triglyceride and LDL-C levels and decreased HDL levels [215]. According to certain research, obese people also have insulin resistance and adverse changes in their glucose metabolism [216,217]. Although it seems that CLA supplementation is widely regarded as safe, certain studies have documented negative effects when CLA (a combination of the two primary isomers, c9, t11, and t10, c12) is taken orally, including fatigue, nausea, diarrhea, and gastrointestinal discomfort [34].

### 6.4. Medium-Chain Triglycerides

Marten et al. [110] reported that MCTs are well tolerated and do not appear to have any significant negative effects. Abdominal cramps, nausea, vomiting, gastrointestinal discomfort, bloating, and osmotic diarrhea are among the unpleasant gastrointestinal symptoms that occur when greater doses of MCTs (>25–30 g) are consumed [218]. Several studies on humans and animals concluded that MCTs had no toxicological properties at levels up to 15% of energy (430 g MCTs per day in a 2000 kcal diet), regardless of whether they were administered parenterally or orally, or if they were consumed as a supplement in a balanced diet [39]. For this reason, it is usually recommended to begin MCT therapy cautiously and increase the dosage gradually.

### 6.5. Phytosterols

When taken in moderation, 1.5–3.0 g of phytosterol-enriched food or supplements is not linked to any significant adverse effects [121]. Consuming phytosterols moderately reduces the absorption of some carotenoids from the gut, bringing their plasma levels near the physiological low end of the oscillation range [120]. A diet high in these chemicals, that is, high in colorful fruits and vegetables, can readily make up for this reduction.

Elevated plasma levels of phytosterols could be associated with an increased risk of cardiovascular events. Nevertheless, it is more likely that the rise in their concentrations in the blood is a sign of an efficient cholesterol absorption pathway, which may be atherogenic, rather than a direct cause of the risk of atherosclerosis [120]. There were no notable adverse effects of phytosterol consumption reported by post-marketing monitoring studies [219,220]. Importantly, dietary phytosterol intake significantly raises cardiovascular risk in patients with homozygous sitosterolemia (in which the ABCG5 and/or ABCG8 transporters are not functional). This syndrome is incredibly rare, affecting only around 1:10,000,000 participants [221].

## 7. Challenges, Limitations, Future Directions, and Recommendations

Most of the studies that have been reviewed contained several limitations that could have had an adverse effect on the results that were reported or made it challenging to reach definitive conclusions. These include the absence of unified research on the development of reliable disease biomarkers and their clear mechanisms of action, reviews of conflict on the ways in which different functional lipids lower the risk of CVDs, insufficient human research, and inconsistent dietary consumption, patterns, doses, efficacy, and long-term consequences in humans. Consequently, further research is necessary to thoroughly understand the dosages, dietary composition, effectiveness, and processes of the many functional lipids that have been discussed. More study is needed to fully comprehend the impact of these functional lipids on human health over the long term, clinical trials, and the development of valid disease biomarkers.

## 8. Conclusions

Due to its health-promoting properties, there has been a notable surge in the demands for functional foods in the last few years. An improper diet and lifestyle are the main causes of the emergence of several health-related disorders. Because fruits, vegetables, meat, and other nutritious goods have anti-inflammatory and antioxidant properties, increasing the intake of these foods can effectively reduce the pathogenicity of various diseases and associated co-morbidities. Healthy dietary components called functional lipids may have an impact on people’s health, reduce their chance of disease, and enhance their quality of life. These health advantages might result in a lower risk of developing CVDs, diabetes, obesity, and depression, among other conditions, and involve modulation of mechanisms and biomarkers associated with the pathogenesis of these conditions. They are inexpensive, readily available, and have been shown to be helpful when incorporated into a diet.

## Figures and Tables

**Table 1:** Dietary sources of functional lipids.

S/N	Functional Lipid	Dietary Sources
1	ω-3 fatty acid (ALA, EPA, and DHA)	Dark green leafy vegetables, flax seed oil, chia seed oil, egg, meat, sea buckthorn, hemp seed oil, canola oil, walnuts, hazelnuts, fatty fish such as mackerel, sardine, tuna, and microalgae.
2	ω-6 fatty acid (GLA and LA)	Black currant oil, evening primrose oil, borage oil, vegetable oil, salad dressing, nuts
3	Conjugated linoleic acid (CLA)	Milk, cultured buttermilk, custard style yogurt, cheddar cheese, meats (kangaroo meat), grass fed ruminants, egg yolk, fish, fresh ground beef, butter fat, plain yogurt.
4	Medium-chain triglyceride (MCTG)	Palm oil, coconut oil, cocoa butter, animal fat.
5	Phytosterols	Brussels, sprouts, flaxseed, peanut butter, cauliflower, olive oil, sesame seeds, Wheat germ, corn oil, canola oil, almonds.

Adapted from Alabdulkarim et al. [25].

**Table 2:** Some clinical studies on CVD health effects of functional lipids.

S/N	Study	Functional Lipids	Population	Dosage (g/d)	Results	References
1	Japan EPA Lipid Intervention Study (JELIS)	EPA	Hypercholesterolemic patients	1.8	The treatment with EPA resulted in a 22% decrease in the CHD Incidence	[144]
2	Randomized clinical trials	*n*-3 PUFA	8179 statin-treated patients with CVD or diabetes and with high TG and LDL-C	4	Reduction of CV events with Icosapent Ethyl-Intervention Trial (REDUCE-IT) showed 25% relative risk reduction in CVD outcomes.	[145]
3	Randomized clinical trials	Phytosterols (stanols)	92 asymptomatic individuals (not using lipid-lowering drugs)	3	Reduction in LDL-C content by 10.2%	[146]
4	Randomized clinical trials	Phytosterol	182 adults with hypercholesterolemia	2	Reduced LDL-C level by 11%	[147]
5	Randomized, double-blind, placebo-controlled trial	EPA	8179 patients	4	The risk of ischemic events, including cardiovascular death, was significantly lowered among those who received icosapent ethyl	[58]
6	Prospective observational	LA	13 countries study (68,659 participants)	High	Higher tissue and circulation concentrations of LA were substantially linked to a lower risk of CV events	[148]
7	A meta-analysis of RCTs	ω-3 PUFA	135,291 subjects.	0.8–1.2	ω-3 PUFA supplementation reduced the risk of major adverse CV events, CV death, and MI	[149]
8	RCTs	Phytosterol (sterols)	30 adults with familial hypercholesterolemia	3	Significantly lower LDL-C by 6.7	[150]
9	RCTs	α-ALA	79 RCTs (112,059 participants)	0.5 to >5	Raising ALA marginally lowered the risk of CVD events, slightly lowered the risk of IHD mortality and arrhythmia	[12]
10	Observational studies and large randomized clinical trails	α-ALA	251,049 individuals and 15,327 CVD events	1.0	ALA intake was associated with reduced risk of mortality, especially CVD mortality. Higher ALA exposure is associated with a moderately lower risk of CVD.	[49]
